# Laparoscopic Distal Pancreatectomy of a Solid Pseudopapillary Tumor (SPT) Achieves Long-Term Oncologic Safety and Multiorgan Preservation

**DOI:** 10.1055/s-0039-1693999

**Published:** 2019-08-21

**Authors:** Ahmed ElHaddad, Paolo Gasparella, Christoph Castellani, Georg Singer, Erich Sorantin, Klara Zach, Holger Till

**Affiliations:** 1 Department of Paediatric and Adolescent Surgery, Medical University Graz, Graz, Austria; 2 Department of Pediatric Surgery, Section of Pediatric Surgery, Tanta University, Tanta, Egypt

**Keywords:** Frantz tumor, solid pseudopapillary tumor, pancreas, oncology, laparoscopy

## Abstract

The oncological safety of a laparoscopic approach for solid pseudopapillary tumors (SPTs) of the pancreas remains a matter of debate. We present the long-term follow-up of an adolescent girl with an SPT in the pancreatic tail. A multimodality workup including magnetic resonance imaging (MRI) revealed a complex, spherical mass of 4.4 cm × 3.6 cm × 4 cm most likely located in the pancreatic tail. All routine laboratory investigations and tumor markers were within normal limits (alpha fetoprotein [AFP], cancer antigen 125 [CA125], CA 19–9, carcinoembryonic antigen [CEA], adrenocorticotropic hormone [ACTH]). Diagnostic laparoscopy was performed to verify the origin of the tumor in the pancreatic tail. In a three-port technique the tumor was mobilized of the splenic vessels until a distal pancreatectomy could be completed. Histopathological examination confirmed the complete resection of a low-grade malignant SPT. The postoperative course was unremarkable. Regular pediatric oncological follow-up examinations for 3 years, including MRI every 6 months, ruled out recurrence and confirmed preservation of splenic and pancreatic functions. While data about the technical feasibility of a laparoscopic approach to pancreatic SPT are already available, this pediatric case report adds a long-term oncological and functional success to the available literature.

## Introduction

Solid pseudopapillary tumors (SPTs) of the pancreas account for 8 to 17% of all pediatric pancreatic neoplasms. 1 2 The tumor was first described by the U.S. American pathologist Virginia Kneeland Frantz in 1959 as a lesion with low-grade malignancy. 3 Malignant behavior is rarely seen and associated with distinct histological findings, such as vascular invasion, peripancreatic infiltration, and lymph node metastases. 4 5 Therefore, complete surgical resection remains the mainstay of successful treatment.

The ideal surgical approach mainly depends on the localization of the tumor. Lesions in the pancreatic body or the tail are usually treated by distal pancreatectomy. The technical feasibility of laparoscopic spleen-preserving distal pancreatectomy for Frantz tumors was first described in 1996. 6 However, few reports in the pediatric population are available about its long-term oncological safety. 7 8

Herein, we present the case of a female adolescent patient with a SPT which was treated laparoscopically with spleen-preserving distal pancreatectomy. Three years of follow-up showed neither recurrences nor signs of metastasis.

## Case Report

A 17-year-old girl was presented to a regional hospital with abdominal pain for several months without emesis or weight loss. Abdominal ultrasonography (US) was performed and revealed a tumorous lesion in the upper abdomen giving the indication for referral to our department.

**Fig. 1:**
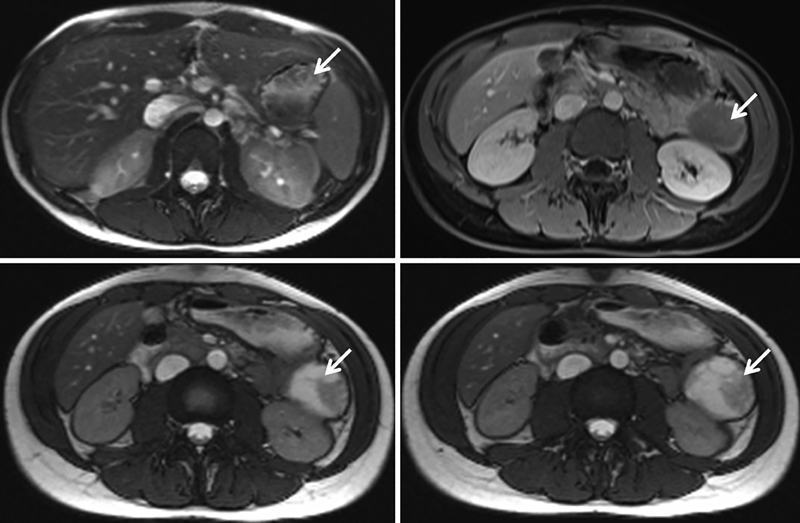
Axial contrast-enhanced MRI of the abdomen showing the tumorous lesion (white arrow) and its relation to adjacent organs. MRI, magnetic resonance imaging.

Upon clinical examination of the patient (body height = 163 cm, body weight = 51 kg), there was slight tenderness in the left upper quadrant without a palpable mass. US was repeated and showed a complex, spherical mass of 4.4 cm × 3.6 cm × 4 cm in diameter with partly complex cystic and solid components. The mass was located between the abdominal wall, the medial margin of the spleen, and the anterior margin of the kidney. Magnetic resonance imaging (MRI) showed a well encapsulated mass at the tail of the pancreas which presented with partially cystic and partially solid areas with contrast enhancement at the periphery of the mass but not in the central cystic part. The lesion was in close relation to the splenic vein superiorly, the medial surface of the spleen laterally, the transverse colon and mesocolon anteriorly, and in front of the left kidney and suprarenal gland. Neither vascular invasions nor lymphadenopathy were detected ( Fig. 1 ). Routine laboratory investigations and tumor markers (alpha fetoprotein [AFP], cancer antigen 125 [CA125], CA 19–9, carcinoembryonic antigen [CEA], and adrenocorticotropic hormone [ACTH]) were within normal ranges.

**Fig. 2:**
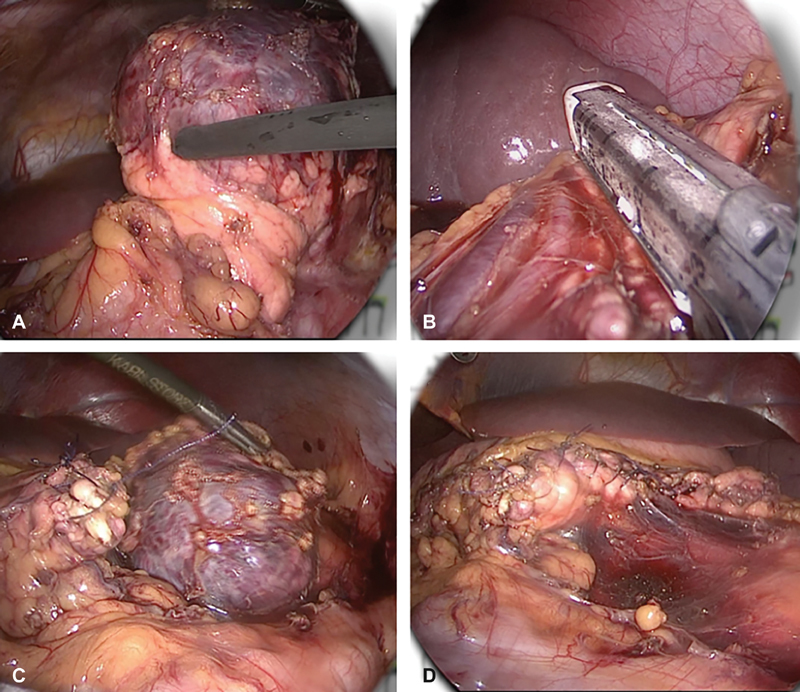
( **A** ) Dissection of the tumor; ( **B** ) distal pancreatectomy using a stapler; ( **C** ) the spherical tumor after complete separation; (D) operative bed with pancreatic stump.

Due to the radiological findings and the negative tumor markers, a solid pseudopapillary tumor of the pancreas was suspected. The decision was taken to proceed with laparoscopy. With the patient in supine position, one 10 mm umbilical trocar and two additional 5 mm trocars in the left- and right-upper abdomen were placed. Laparoscopic distal pancreatectomy with preservation of the spleen was done using a 30-degree camera ( Fig. 2 ). Dissection was assisted with LigaSure and separation of the mass from the pancreas using Endo GIA Auto Suture Universal Stapler (Covidien, Minneapolis, MN, United States). A retrieval bag was used for removal of the mass. Operation time was 165 minutes.

Histopathological examination revealed a completely resected pseudopapillary tumor of the pancreas with 5.5 cm in diameter. The proliferation fraction (Ki67 staining) within the tumor tissue was below 5%. Immunohistochemical examination was negative for keratin, chromogranin, CEA, and S-100 and positive for (neuron specific enolase) NSE, β-catenin, and α-1 antitrypsin. Additionally, single cells were positive for vimentin und synaptophysin.

The postoperative course was uneventful except for a temporary peripancreatic fluid collection managed conservatively. Repeated ultrasound examinations were performed until complete resolution without pseudopancreatic cyst formation was seen 7 months postoperatively.

Sonographic and clinical follow-up examinations, every 3 months during the first year and every 6 months thereafter, were unremarkable. In addition, MRI was performed twice a year in the first 2 postoperative years and at the end of the third year without evidence of a residual tumor or a recurrence. Moreover, body weight, as well as endocrine and exocrine function of the pancreas, was normal 3 years following removal of the tumor.

## Discussion

SPTs, also termed as Frantz tumors, are rare low-grade malignant pancreatic tumors that typically occur in females aged between 20 and 40 years. 8 The present case describes an adolescent female with a SPT located in the pancreatic tail which was managed by laparoscopic spleen preserving distal pancreatectomy. Neither complications nor recurrence were encountered during 3 years of follow-up.

Due to the rarity of these lesions, reports describing SPT of the pancreas only including pediatric patients are confined to case reports or case series. 8 9 Crocoli and coworkers have recently reported one of the largest series including detailed information of 43 pediatric patients with SPT. 10 The authors have found the typical female preponderance and the median age at diagnosis was 13.2 years.

The clinical presentation is variable and ranges between asymptomatic incidental discovery, abdominal discomfort and pain, palpable mass, nausea, vomiting, and weight loss. 8 However, there seems to be a difference in the clinical manifestation between children and adults. In an article published in 2008, clinical features of 15 pediatric cases were compared with 47 adult patients. 11 In adults, the diagnosis was usually made incidentally during screening by detection of a mass. However, all of the included children were symptomatic. 11 In contrast, 21% of the children described in another study, by Crocoli et al, had an incidental diagnosis and the remaining patients were diagnosed on abdominal ultrasound performed due to abdominal pain or had a palpable mass. 10 A rare case with hemoperitoneum due to traumatic tumor rupture has also been described in the literature. 12 Taken together, prompt abdominal ultrasound in pediatric patients with unspecific abdominal pain seems to be a prerequisite for rapid diagnosis.

The tumor can arise from any part of the pancreas (head, body, or tail). Moreover, rare cases of extrapancreatic manifestations, such as the adrenal gland, have been reported. 13 Regarding the most prevalent localization of the tumor in the pancreas, there are contradictory findings in the literature. While some reports have found that the body/tail was most often affected, 10 others describe the pancreatic head as the most common localization 11 or an even distribution between head and tail. 8 In our patient, the mass arose from the tail of the pancreas in close relation to the spleen, left kidney, and adrenal gland.

US examinations of SPTs usually show a homogeneous, hypoechogenic mass with a hyerpechogenic rim. 14 Computed tomography features include an encapsulated mass with varying solid and cystic components secondary to hemorrhagic degeneration. 14 At the periphery of the mass, both solid areas and calcifications can be seen. 15 Typical MRI findings are a large, well-defined, encapsulated lesion with heterogeneous high-or low-signal intensity on T1-weighted and heterogeneous high signal intensity on T2-weighted images. 16

The use of preoperative biopsies is discussed controversially in the literature. While it may be useful in verifying the diagnosis 17 it could result in tumor dissemination in case of malignancy. 18 Additionally, it has been shown that biopsies have limited diagnostic accuracy of 56%. 19 This is mirrored by a comprehensive review of 718 patients where only 2.7% underwent biopsies. 2

To achieve excellent long-term outcome, surgery is the mainstay of SPT treatment. Since local recurrences and metastases are reported following incomplete resection, 4 5 20 complete resection of the tumor with preservation of as much pancreatic tissue as possible is warranted. The ideal surgical approach depends on the localization of the lesion and consists of pancreaticoduodenectomy with or without pyloric preservation for lesions located in the pancreatic head and of distal pancreatectomy with or without splenectomy for lesions in the pancreatic body or tail. 8 Moreover, cases of duodenum preserving pancreatic head resections and resection of the tumors have been described. 1 21

The use of minimally invasive surgery (MIS) for oncological procedures is debated in the literature due to the risk of tumor rupture and subsequent spillage. For SPT, Coelho and coworkers have reported 20 patients with SPT of whom 7 underwent laparoscopic resection with one intraoperative tumor rupture. 22 Crocoli et al described a series of 43 children with one case of tumor rupture (spillage). 10 However, it is unclear whether this patient was treated by open surgery or MIS.

Nevertheless, laparoscopic procedures, like distal pancreatectomy, have been considered safe and feasible for SPT. 7 23 Recently, also a case of robotic pylorus preserving pancreatoduodenectomy for a solid pseudopapillary tumor of the pancreas in a 10-year-old child has been published. 24 In our case, the tumor had the characteristic imaging of SPT without signs of lymphadenopathy or invasion of adjacent vessels or organs. Therefore, the decision for a minimally invasive approach was made. Meticulous follow-up examinations consisting of clinical examination, ultrasound, and MRI did not show any signs of recurrence in our patient, 3 years postoperatively.

## Conclusion

In conclusion, laparoscopy in an adolescent patient with an SPT allowed complete resection following basic oncological principles, preservation of splenic and pancreatic function, and avoidance a major laparotomy. While data about the technical feasibility of a laparoscopic approach to pancreatic SPT are already available, this pediatric case report adds a long-term oncological and functional success to the available literature.

## Notes

New Insights and the Importance for the Pediatric Surgeon Laparoscopy in an adolescent patient with a solid pseudopapillary tumor located in the pancreatic tail allowed complete resection following basic oncological principles, preservation of splenic and pancreatic function, and avoidance a major laparotomy.
